# Detailed Per-residue Energetic Analysis Explains the Driving Force for Microtubule Disassembly

**DOI:** 10.1371/journal.pcbi.1004313

**Published:** 2015-06-01

**Authors:** Ahmed T. Ayoub, Mariusz Klobukowski, Jack A. Tuszynski

**Affiliations:** 1 Department of Chemistry, University of Alberta, Edmonton, Alberta, Canada; 2 Department of Oncology, University of Alberta, Edmonton, Alberta, Canada; 3 Department of Physics, University of Alberta, Edmonton, Alberta, Canada; University of Houston, UNITED STATES

## Abstract

Microtubules are long filamentous hollow cylinders whose surfaces form lattice structures of *αβ*-tubulin heterodimers. They perform multiple physiological roles in eukaryotic cells and are targets for therapeutic interventions. In our study, we carried out all-atom molecular dynamics simulations for arbitrarily long microtubules that have either GDP or GTP molecules in the E-site of β-tubulin. A detailed energy balance of the MM/GBSA inter-dimer interaction energy per residue contributing to the overall lateral and longitudinal structural stability was performed. The obtained results identified the key residues and tubulin domains according to their energetic contributions. They also identified the molecular forces that drive microtubule disassembly. At the tip of the plus end of the microtubule, the uneven distribution of longitudinal interaction energies within a protofilament generates a torque that bends tubulin outwardly with respect to the cylinder's axis causing disassembly. In the presence of GTP, this torque is opposed by lateral interactions that prevent outward curling, thus stabilizing the whole microtubule. Once GTP hydrolysis reaches the tip of the microtubule (lateral cap), lateral interactions become much weaker, allowing tubulin dimers to bend outwards, causing disassembly. The role of magnesium in the process of outward curling has also been demonstrated. This study also showed that the microtubule seam is the most energetically labile inter-dimer interface and could serve as a trigger point for disassembly. Based on a detailed balance of the energetic contributions per amino acid residue in the microtubule, numerous other analyses could be performed to give additional insights into the properties of microtubule dynamic instability.

## Author Summary

The molecular machinery of chromosome segregation during cell division is one of the most sophisticated molecular biology mechanisms employing the interplay of different proteins and forces. The long filamentous tube-shaped microtubule structure is a central player in chromosome segregation and cell division, making it an important physiological and therapeutic target. However, the driving force for microtubule disassembly and dynamic instability, and hence force generation, is still not fully understood. In our all-atom molecular dynamics simulations we calculated the energy of interactions, within a microtubule cylinder, that is responsible for microtubule stability. We broke this energy down to individual contributions of every residue and domain. Different energy profiles enabled us to unravel the driving force behind microtubule disassembly and force generation, a longstanding unanswered biological question. We also elucidated the mechanism of disassembly and explained the effects of different factors on disassembly rates. Our list of energetic contribution of single amino acid residues could also serve in tailor-designing engineered microtubules that could be used for therapeutic and diagnostic purposes.

## Introduction

Microtubules (MTs) are cellular organelles that participate in major cellular processes such as mitosis, cell shape maintenance, cell motility and motor protein transport and constitute a major target for a wide range of drugs, most notably anti-mitotic chemotherapy agents such as paclitaxel. Due to their importance in cell biology, MTs have been the topic of active research into their structure and function for several decades [1]. The pivotal role of MTs in cell division, by forming the mitotic spindle that segregates chromosomes, makes them an important target for antimitotic cancer chemotherapy drugs [2, 3].

**Fig 1:**
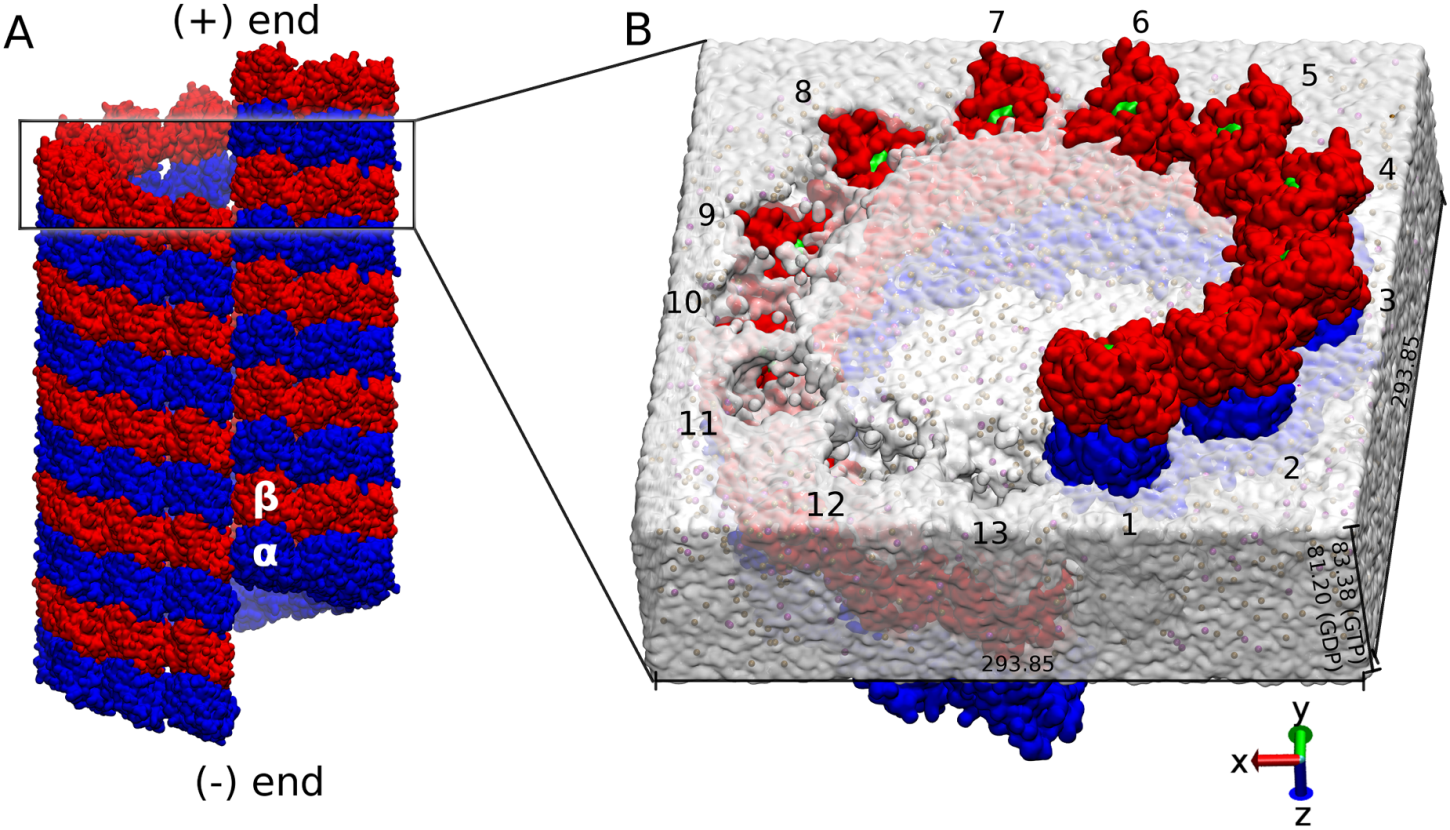
Model of MT structure. (A) A model of an MT lattice showing *α* (blue) and *β* (red) tubulin subunits. It shows the plus and minus end as well as the seam. (B) A model of the system used in the molecular dynamics simulations. Tubulin dimers are numbered from 1 to 13, GDP (or GTP) cofactor is shown in green within *β*-tubulin, the second GTP cofactor is buried between *α* and *β* subunits, water is represented by the white box, within which purple spheres represent Cl^−^ and brown spheres represent Na^+^. Periodic box dimensions in units of Å are also shown.

The peanut-shaped *αβ*-tubulin heterodimer is the building block of MTs [4]. Tubulin heterodimers associate longitudinally to form protofilaments, which in turn associate laterally to form a left-handed three-start helix with a seam, that results in the most common microtubule structure, the so-called B lattice [5]. Since tubulin dimers polymerize end to end, MTs become polarized, meaning that one end has *α*-subunits exposed (minus end) while the other end where faster growth usually occurs has *β*-subunits exposed (plus end) (Fig 1A) [6]. Within a tubulin heterodimer, GTP binds at the *α*-tubulin N-site which occurs at the intra-dimer interface. This GTP molecule does not undergo hydrolysis. Another GTP molecule attaches at the *β*-tubulin E-site and undergoes hydrolysis to GDP and phosphate shortly after assembly [7], in a process which drives the stochastic switching between growth and shrinkage in MTs. This unique property of microtubules is commonly referred to as dynamic instability [8]. Mitchison and Kirschner proposed the so-called GTP-cap model, which states that as long as the plus end of an MT is capped with GTP, it continues to grow. However, if GTP hydrolysis is sufficiently fast to catch up to the growing tip of the MT, rapid shrinkage, called a catastrophe, results [9]. Upon binding to an MT, some pharmacological agents such as taxol or epothilone stabilize the system and inhibit shrinkage [10]. Several studies have been conducted to determine which specific structural transitions that accompany GTP hydrolysis or taxol binding are responsible for their effect on MT stability, especially the transition of the tubulin dimer between its straight and curved states [11–15]. In the most recent of these studies, Alushin et al. found that GTP hydrolysis leads to a compaction around the E-site nucleotide which is reversed upon taxol binding [15]. This compaction was proposed to generate a strain which is powered by the energy of GTP hydrolysis and is believed to be released only through outward curving of protofilaments, initiating disassembly [16]. A missing component in these studies, however, is the quantification of the free energy changes that accompany these structural transitions. Due to the difficulties related to its experimental measurements, many simulations have been conducted to study detailed MT energetics [17–22]. In a recent study we have analyzed the strength of hydrogen bonds that bring and hold tubulin subunits together within different lattice configurations [23]. However, in all of these simulations, several factors were still missing. Most importantly, the full energetics of a complete MT model, which is essential to understanding the thermodynamics of tubulin assembly, has not been estimated yet due to the high computational price associated with such analyses. A detailed energy balance involving contributions due to each residue, domain or subunit, to the best of our knowledge, was never considered.

As a result of recent advances in computational technology, GPU-based computations can now be implemented to perform very demanding calculations in a reasonable amount of time. With this technology readily available, we simulated two complete all-atom MT models and studied in detail their energetics. The models studied are: (a) an MT with GDP in the E-site (GDP-Model) and (b) an MT with GTP in the E-site (GTP-Model). We did not need to look for a non-hydrolyzable analogue of GTP as hydrolysis is not a problem in molecular dynamics simulations, in contrast to experimental procedures [16]. The MT model that we used was initially built by Wells and Aksimentiev [24] utilizing sophisticated theoretical techniques to combine experimental structural information from a cryo-electron microscopy map of MT at 8 Å resolution [25] and electron crystallography structure of tubulin at 3.5 Å resolution [26]. We combined this model with the recently published crystal structures [15] in order to generate an atomistic representation involving an infinite number of infinitely long MTs. This is possible due to the use of periodic boundary conditions. (see S1 Movie).

## Results

### Molecular Dynamics Equilibration

**Fig 2:**
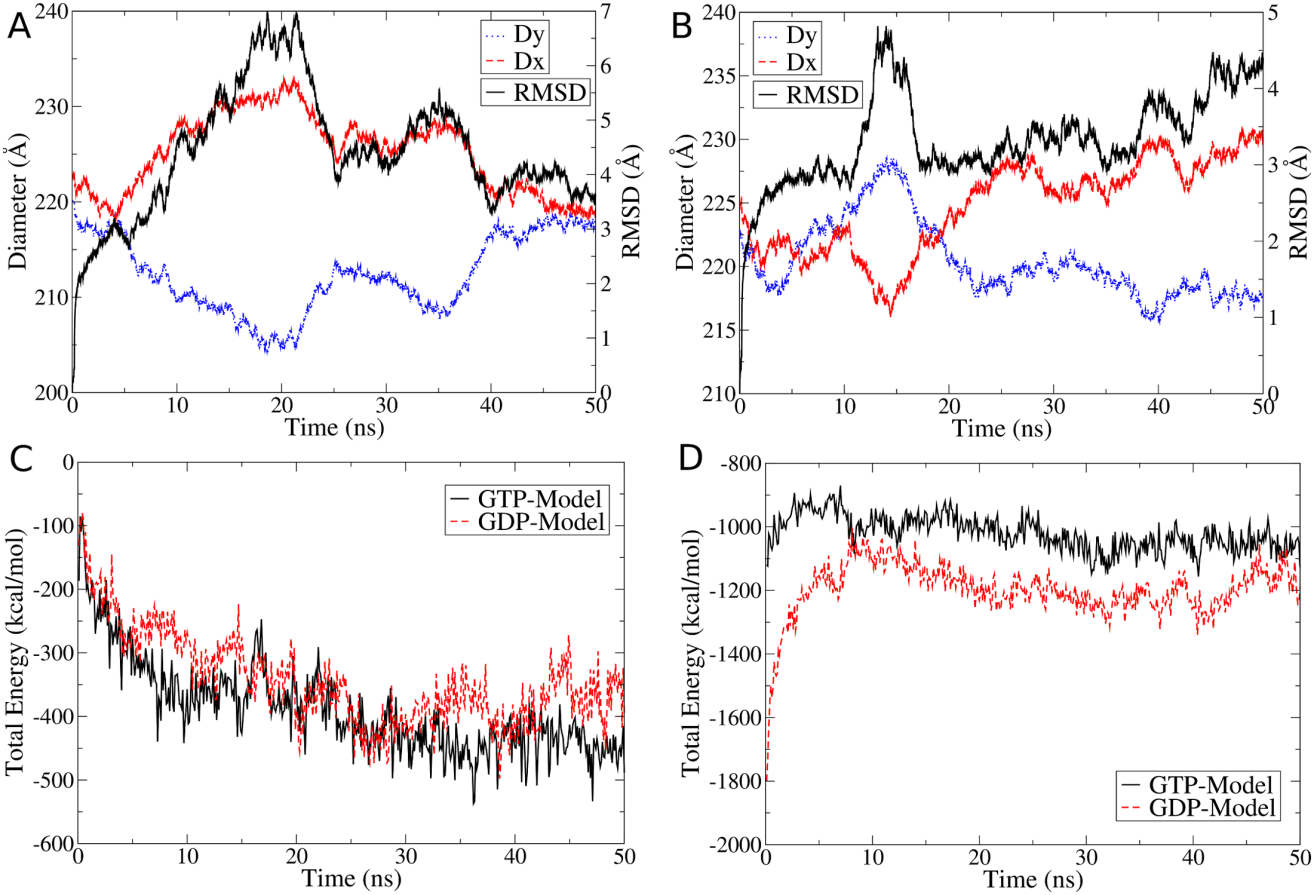
Equilibration plots. Plots showing the change in RMSD of protein backbone atoms and the two nearly perpendicular diameters *Dx* and *Dy* over simulation time in (A) GTP-Model and (B) GDP-Models. Equilibration of total sum of interaction energies versus simulation time across (C) lateral and (D) longitudinal inter-dimer interfaces.

A 50-ns MD trajectory was analyzed for several equilibration aspects, the first of which is the root-mean-square deviation (RMSD) of the backbone atoms relative to the starting structure. In addition, two nearly perpendicular MT cylinder diameters, namely *Dx* and *Dy*, were also calculated along the trajectory. Referring to the tubulin dimer numbering in Fig 1B, the diameter *Dx* was defined as the distance between the center of mass of dimer 4 and the center of mass of dimer 10 and 11, while *Dy* was defined as the distance between the center of mass of dimer 1 and the center of mass of dimer 7 and 8. In both diameters, only the distance projection on the *x*-*y* plane was considered as this is what gives the cylinder diameter. Plots showing the change in RMSD of the backbone atoms, *Dx* and *Dy* over simulation time for the GDP- and GTP-Models are shown in Fig 2A and 2B. The two diagrams indicate a strong correlation between fluctuations in RMSD and in diameters which indicates that most of RMSD fluctuations are due to changes in the circular shape of MT cylinders rather than the rearrangement of domains. The two diagrams also show the flexibility of MT cylinders as they deform spontaneously from a circular to an oval shape and vice versa. Movies showing the change of the two diameters over simulation time can be found in Supporting Information (see S2 and S3 Movies).

Since our particular interest is in the MT energetics, we used the overall MT energy across lateral and longitudinal inter-dimer interfaces as an indication of whether the system is equilibrated or not. Hence, we calculated these energies using MM/GBSA and the formula in Eqs 1 and 2 and plotted the total energy per MT ring versus simulation time (Fig 2C and 2D). Both plots indicate that the overall lateral and longitudinal energies in both the GDP- and GTP-Models have already equilibrated at least before the last 20 ns of the MD simulation time. The plots also show that the large fluctuations in RMSD or *Dx* and *Dy* hardly affect the MT energetics at either of the two interfaces, which is a good indication of the energetic stability of our models.

### Lateral Energetics in the GDP-Model

Total breakdown of the predicted energy contributions enabled us to perform the analysis for different residues, domains, subunits, and dimers across both lateral and longitudinal inter-dimer interfaces. Before listing the results, it should be noted that energies calculated via the MM/GBSA method do not necessarily reflect absolute energy values. Rather, they are used for relative comparison within the same model [27]. It should also be noted that all energies listed here are calculated per MT ring, unless otherwise specified.

**Fig 3:**
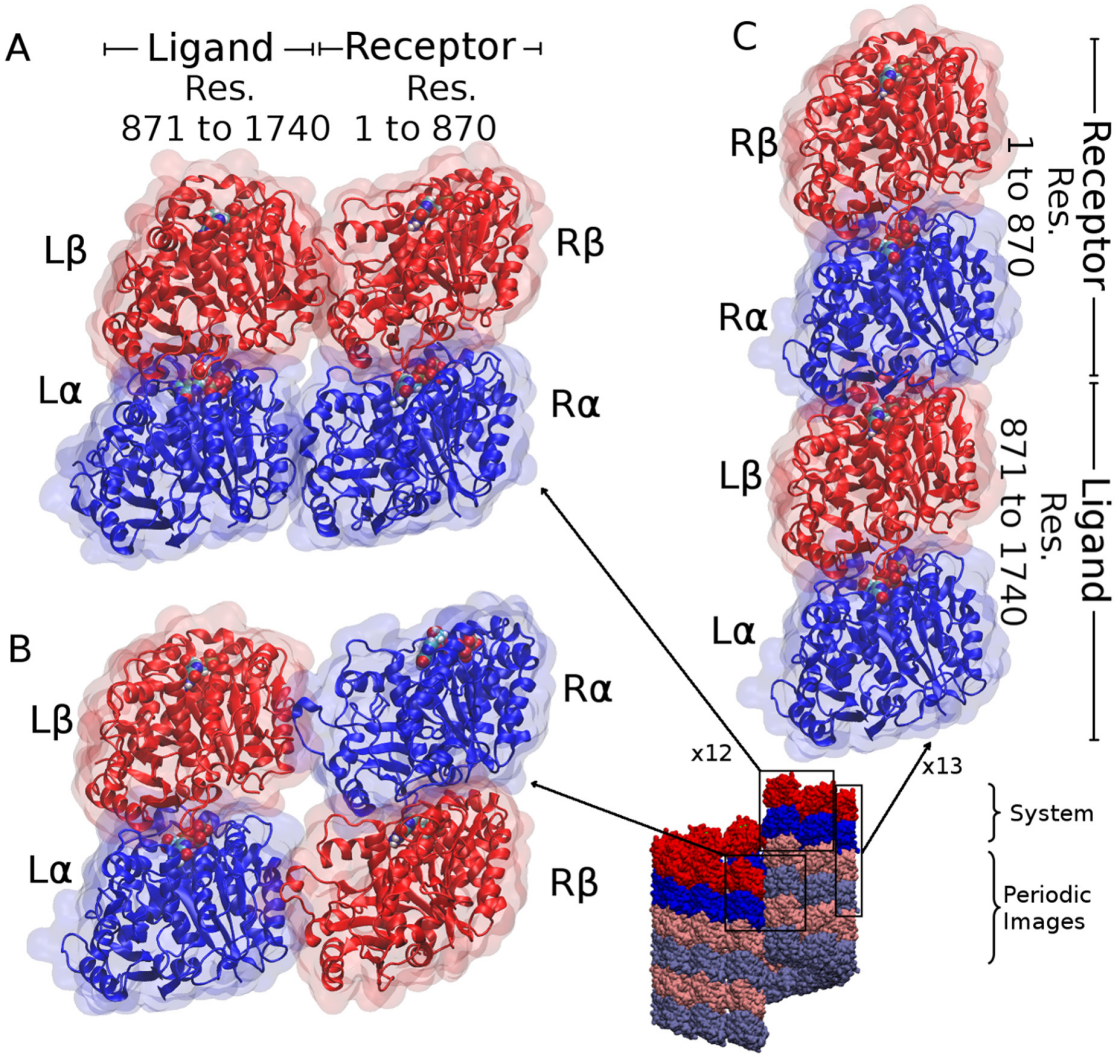
Tubulin subsystems used for MM/GBSA calculations. (A) A subsystem of two lateral tubulin dimers extracted from MT simulations where the receptor (*R*) is tubulin *k* and the ligand (*L*) is tubulin *k*+1, where *k* runs from 1 to 12, (B) Same as (A) but at the seam. I.e. the receptor is tubulin 13 and the ligand is the periodic image of tubulin 1. Residue (Res.) numbering in (A) and (B) are the same, (C) A subsystem of two longitudinal tubulin dimers where the receptor is tubulin *k* and the ligand is its periodic image, where *k* runs from 1 to 13. Total number of residues may differ slightly between the GDP and GTP models.

As Table 1 summarizes, the overall energy of interaction across the 13 lateral tubulin interfaces (see Fig 1B), $E_{tot}^{lat}$, was found to be −411±29 kcal/mol, nearly 60% of which is due to *α*-*α* interactions and the rest is due to *β*-*β* interactions. On the other hand, the contribution of the dimer acting as a receptor (see the explanation of the ligand/receptor convention in the methods section and in Fig 3A and 3B), $E_{R}^{lat}$, was about 54% of the overall energy while the rest was attributed to the ligand, $E_{L}^{lat}$, with the difference entirely attributed to solvation effects rather than direct interactions. It should be noted, however, that the *α* subunit of the ligand (L*α*) and the *β* subunit of the receptor (R*β*) together contribute −312±29 kcal/mol which is nearly 75% of $E_{tot}^{lat}$, with the L*α* contribution slightly larger than that due to R*β*. The contribution of L*β* and R*α* was found to be much smaller, only 25% of $E_{tot}^{lat}$. Upon structural inspection, this 50% difference, being almost entirely due to electrostatic interactions, was attributed to diagonal interactions between subunits; although the interface between L*α* and R*β* is dominated by oppositely-charged residues and thus stabilizing the interaction, the opposite is true at the destabilizing interface between R*α* and L*β* which has, for example, residues R*α*/Glu220 and L*β*/Asp130 destabilizing the lateral interface by 12±1 and 10±2 kcal/mol, respectively.

**Table 1:** A matrix showing individual contributions of each subunit to lateral stability in the two simulated systems, in kcal/mol.

	**GDP-Model**			**GTP-Model**		
Subunit	L	R	Tot. (L+R)	L	R	Tot. (L+R)
*β*	−25±14	−147±15	−172±20	−61±14	−145±16	−206±21
*α*	−165±14	−74±15	−239±20	−193±15	−83±14	−276±20
Tot. (*β*+*α*)	−190±19	−221±21	−411±29	−254±20	−228±21	−482±29

Cell “L*β*”, for example, refers to the contribution of the *β* subunit of the dimer acting as ligand.

As to the energetic breakdown according to interaction types, the contribution of the van der Waals and non-polar solvation energy, *E*(vdW+SA), to the overall energy is largely stabilizing with an average value of −1476 kcal/mol, 85% of which is due to the vdW interactions. This stabilization is opposed by destabilization due to electrostatic interactions; the average sum of electrostatic and the polar solvation energy, *E*(ele+GB), is 1065 kcal/mol. This is expected since tubulin dimers are highly negatively charged and tend to repel each other.

Regarding the detailed energy contributions per individual residues, the most important residue across the lateral interface was found to be R*β*/Tyr283 followed by R*α*/His283 and L*α*/His88, with overall stabilization energies of −90±5, −47±5 and −42±3 kcal/mol per MT ring, respectively. R*β*/Tyr283 alone supplies more than 20% of lateral stability most of which is due to the vdW interactions. In fact, most of the stabilizing residues on top of our list were neutral ones with a strong stabilizing vdW component. On the other hand, almost all of the destabilizing residues were charged ones with a strong electrostatic component, most destabilizing of which is L*β*/Lys124 with an energy of 22±7 kcal/mol. A complete list of the different energetic contributions of each residue in the ligand and receptor per MT ring is provided in the Supporting Information.

**Fig 4:**
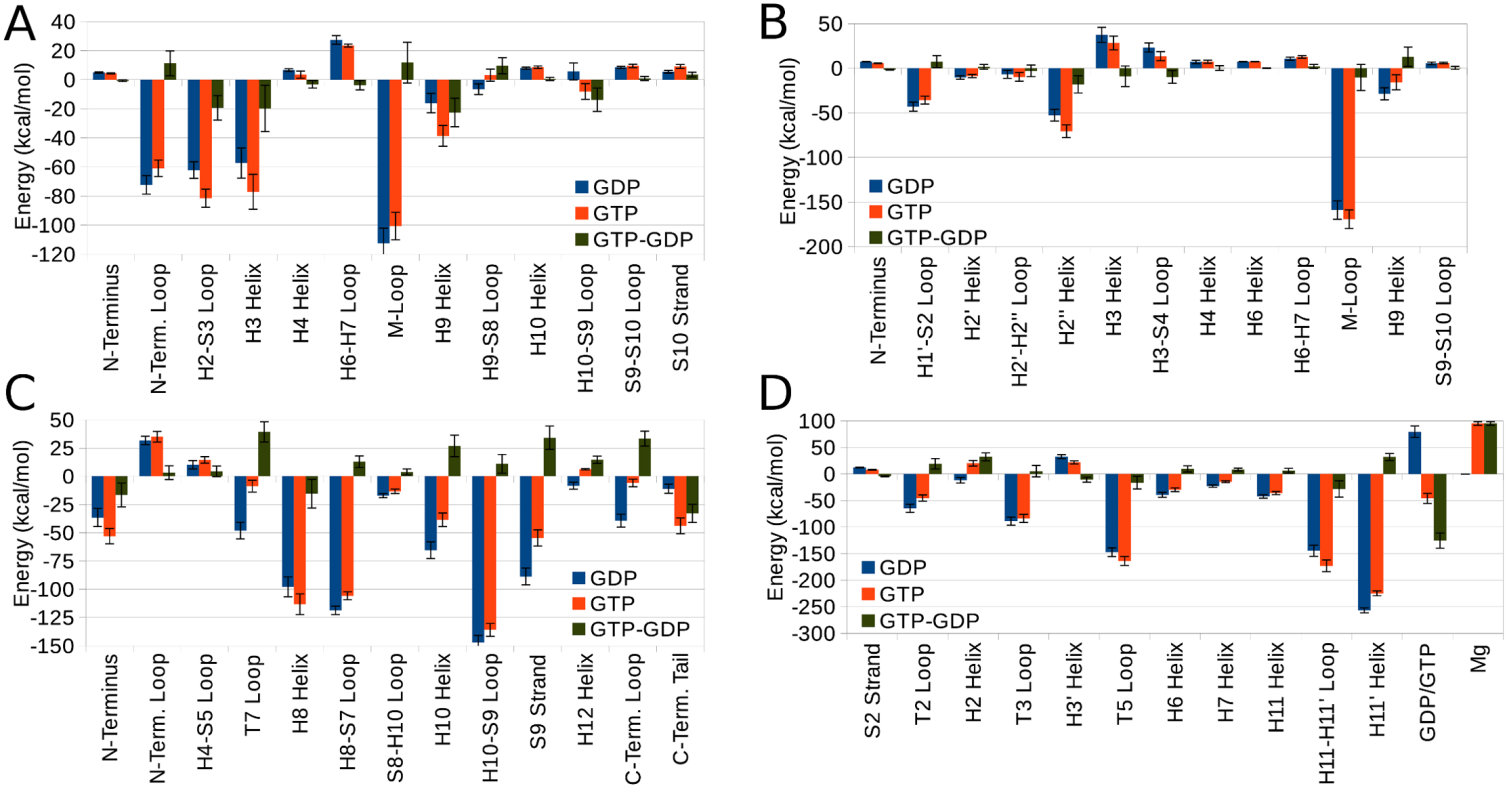
Domain contributions to overall energy. Energetic contributions of important domains across lateral interface in (A) *α* and (B) *β* subunits and across longitudinal inter-dimer interface in (C) *α* and (D) *β* subunits. Data are shown for GDP- and GTP-Model as well as the difference between them (GTP-GDP). On the *x*-axis of (A) and (B), domains H4 helix and before occur at lateral interface of the ligand while domains after that occur at receptor lateral interface. In (C), all domains belong to receptor while all the domains in (D) belong to ligand. See ligand and receptor definitions in Fig 3.

Domain contributions to the overall energy per MT ring were also calculated and Fig 4A and 4B show the most relevant of them. The contribution of the M-loop in both *α* and *β* subunits is by far the largest, with values of −112±10 and −159±10 kcal/mol, respectively, making up about two thirds of the energy of the overall lateral interactions. This agrees well with previous predictions, although precise values of their energetic contributions were never calculated [25, 28, 29]. Other less important domains are the L*α*/N-terminal loop, L*α*/H2-S3 loop, L*α*/H3 helix and L*α*/H9 helix at the *α* interface with a stabilization of −72±6, −62±6, −57±10 and −16±7 kcal/mol, respectively [25, 28]. L*β*/H3 helix at the *β*-*β* interface, however, has a strongly destabilizing effect of 37±8 kcal/mol. This supports previous predictions based on structural analysis by Li et al. and Nogales et al; however, these authors did not specify if these interactions are stabilizing or not [25, 28]. Additionally, L*β*/H2” helix and L*β*/H1’-S2 loop also have relatively strong stabilizing contributions of −53±7 and −43±5 kcal/mol, respectively.

### Lateral Energetics in the GTP-Model

As Table 1 summarizes, the overall interaction energy across the lateral interface in the GTP-Model, $E_{tot}^{lat}$, was found to be −482±29 kcal/mol, nearly 60% of which is due to the *α*-*α* interactions. This average overall energy is 71 kcal/mol (nearly 20%) more stable than the overall energy of the GDP-Model which explains the role of GTP in stabilizing MTs as will be shown later. Nearly 90% of this difference in stability is solely attributed to enhancement of the contribution of the ligand, both *α*- and *β*-subunits, rather than the receptor. As was noticed in the GDP-Model, L*α* and R*β* are also responsible for most of the lateral stabilization in the GTP-Model, −338±22 kcal/mol (70% of $E_{tot}^{lat}$).

**Fig 5:**
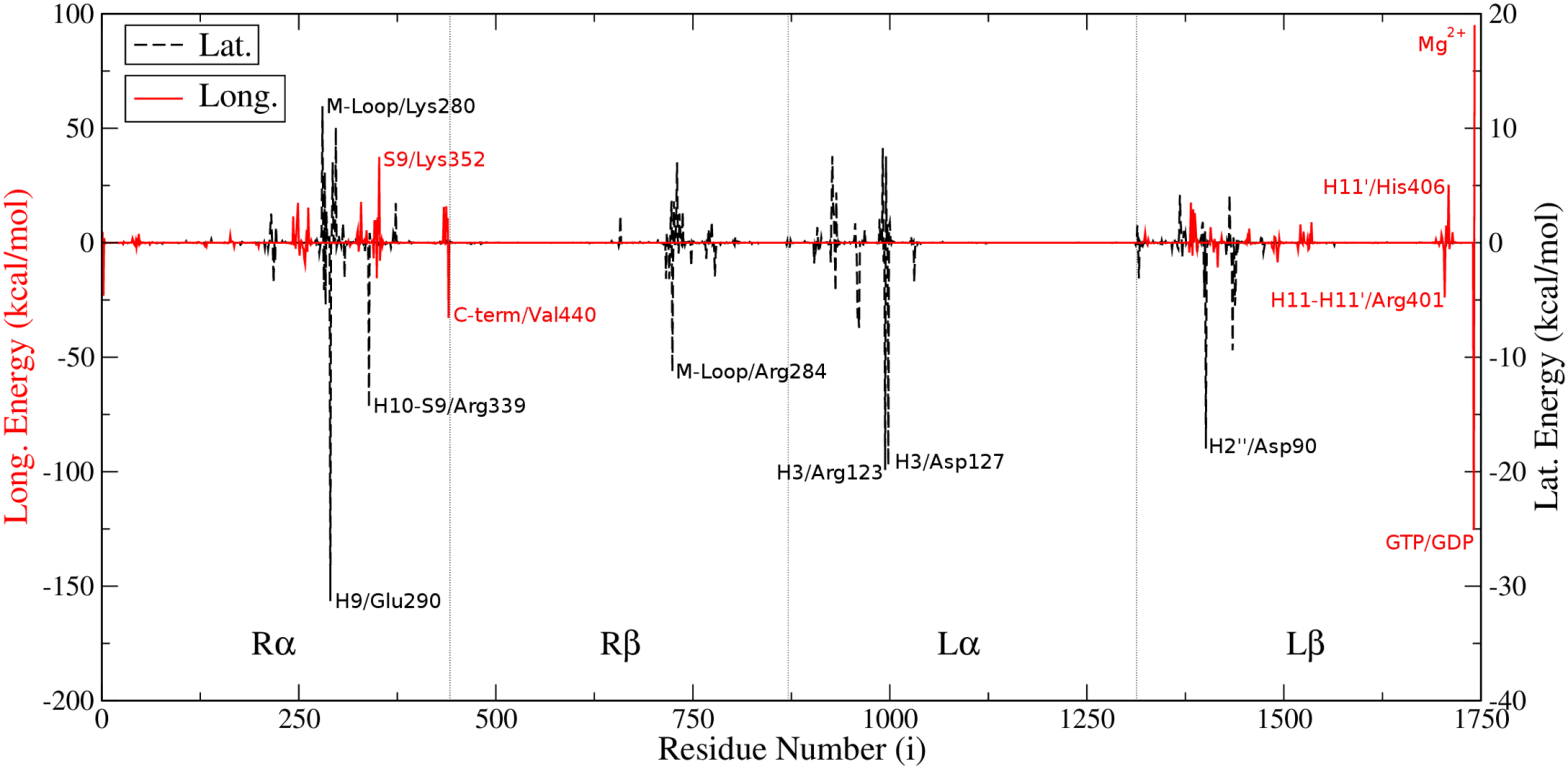
Energetic contributions of residues. Difference between overall residual contributions per MT ring in GTP- and GDP-Model; ($E_{i}^{\text{GTP}}-E_{i}^{\text{GDP}}$), where *i* is the residue number running from 1 to 1742. Different energy axes are used due to differences in magnitude of interactions at both interfaces. Important residues are labeled together with their domains.

Upon breakdown of the interaction energy to its individual components, we find that in the GTP-Model, the *E*(vdW+SA) contribution becomes −1432 kcal/mol while *E*(ele+GB) becomes 950 kcal/mol. Comparing this to the GDP-Model, it turns out that GTP destabilizes the vdW and non-polar solvation interactions by 44 kcal/mol and stabilizes electrostatic and polar solvation interactions by 115 kcal/mol, which results in the net stabilization of 71 kcal/mol as mentioned earlier. This difference becomes clear by analyzing Fig 4A and 4B for domain contributions and Fig 5 for residual contributions. It is apparent from Fig 4A that GTP strengthens the contributions of the L*α*/H3 helix and R*α*/H9 helix by 23±10 and 20±16 kcal/mol, respectively. Most of this helix stabilization can be attributed to interactions involving R*α*/Glu290 (residue number in Fig 5, *i*, is 290), residue L*α*/Asp127 (*i* = 998), and residue L*α*/Arg123 (*i* = 994). These three residues stabilize the GTP-Model over the GDP-Model by energy values of 31, 20 and 19 kcal/mol, respectively, mostly due to electrostatic interactions. Upon structural analysis it is apparent that GTP slightly rotates the dimer acting as a ligand toward the one acting as a receptor, thus allowing stronger interactions between H3 and H9 helices with oppositely-charged residues. GTP also enhances the stability imparted by the L*α*/H2-S3 loop and the R*α*/H10-S9 loop, although it moderately decreases the role of the L*α*/N-terminal loop as well as the R*α*/M-loop in the overall MT stability.

Similar conclusions are reached in regard to the *β*-subunit and the effect of the L*β*/H2” helix through residue L*β*/Asp90 (*i* = 1401) and the R*β*/M-loop through residue R*β*/Arg284 (*i* = 724). Both domains are stabilized in the GTP-Model by extra 18±10 and 10±15 kcal/mol compared to the GDP-Model, respectively. The charged nature of all these residues explains why most of GTP stabilization is manifested in *E*(ele+GB) not *E*(vdW+SA). Fig 4B also shows that GTP reduces the destabilization caused by the L*β*/H3 helix and the L*β*/H3-S4 loop. On the other hand, GTP reduces stability imparted by the L*β*/H1’-S2 loop and the R*β*/H9 helix. Details of the contribution of each residue in the GTP-Model can be found in the Supporting Information.

### Longitudinal Energetics in the GDP-Model

Analysis of the strength of interactions across the longitudinal inter-dimer interface in the GDP-Model yielded, as summarized in Table 2, an overall energy of −1240±32 kcal/mol per MT ring, which is nearly three times the lateral interaction energy. This is in agreement with structural observations [28]. Due to the orientation of tubulin dimers at the longitudinal inter-dimer interface, the contributions of L*α* and R*β* are essentially zero and will always be neglected here. On the other hand, the contribution of L*β* is 54% of the total value, and the remainder is contributed by R*α*. The breakdown of this energy yields an average *E*(vdW+SA) of −2668 kcal/mol which is almost twice as large as the value across the lateral interface. This is obviously due to the tighter packing of the residues here as opposed to looser packing at the lateral interface. The average *E*(ele+GB) across the longitudinal inter-dimer interface is 1428 kcal/mol and it is 34% larger than its value at the lateral interface.

**Table 2:** A matrix showing individual contributions of each subunit to longitudinal stability in the two simulated systems, in kcal/mol.

	**GDP-Model**			**GTP-Model**		
Subunit	L	R	Tot. (L+R)	L	R	Tot. (L+R)
*β*	−664±24	0	−664±24	−623±22	0	−623±22
*α*	0	−576±21	−576±21	0	−475±20	−475±20
Tot. (*β*+*α*)	−664±24	−576±21	−1240±32	−623±22	−475±20	−1098±30

Per-residue energy analysis reveals the most important residues to longitudinal stability, the first of which is L*β*/Arg401 from the H11-H11’ loop which alone supplies −101±7 kcal/mol (nearly 10%) [23]. After that come residues L*β*/Phe404 and L*β*/Trp407 from the H11’ helix both of which support longitudinal stability by contributing −91±3 and −78±3 kcal/mol, respectively. This makes the two former domains, which constitute part of the tubulin C-terminal domain, the most critical for longitudinal stability in the *β*-subunit (Fig 4D). The figure also shows that the following domains in the L*β* subunit: the T5 loop, T3 loop, and T2 loop are also very important for longitudinal stability. The role of the GDP cofactor appears quite influential at the longitudinal inter-dimer interface, in contrast to the lateral one. It is primarily destabilizing with a large contribution of 79±11 kcal/mol due mainly to a strong electrostatic repulsion with the highly negative environment, despite its strong salt bridge with R*α*/Lys352. Residual analysis of the R*α* subunit also shows some relatively less important residues; R*α*/Trp346, R*α*/Tyr262 and R*α*/Lys352 with energy contributions of nearly −60 kcal/mol for each of them. These and other residues are responsible for the following domains in the R*α* subunit: the H10-S9 loop, H8-S7 loop, and the S9 strand being the top stabilizers in Fig 4C. The R*α*/H8 and R*α*/H10 helices are also relatively important for longitudinal stability. Both the R*α*/C- and R*α*/N-terminal domains are important as well, with the R*α*/N-terminal loop being a destabilizer, in contrast to its role at the lateral interface.

### Longitudinal Energetics in the GTP-Model

As summarized in Table 2, the overall interaction energy across the longitudinal inter-dimer interface in the GTP-Model was found to be −1098±30 kcal/mol per MT ring, which is 141 kcal/mol (10%) less stable than the GDP-Model system. This difference is attributed to a 7% decrease in the R*α* and 3% decrease in the L*β* interactions. Upon energetic breakdown we see that GTP destabilizes the vdW and non-polar solvation energy by nearly 250 kcal/mol, while stabilizing electrostatic and polar solvation energy by nearly 110 kcal/mol. This could be due to the longstanding observation that GTP leads to an expansion in the E-site and lengthening of the tubulin dimers. That is, axial dimer repeat changes from 81.20 Å in GDP-tubulin to 83.38 Å in GTP-tubulin [12, 15]. This reduces the packing of atoms at the interface and hence lowers both the vdW attraction and electrostatic repulsion, the former being affected most due to its stronger dependence on distance.

Looking into domain contributions in Fig 4C and 4D we see how GTP destabilization of longitudinal interactions can be subdivided. The most pronounced difference between the GDP- and the GTP-Model appears in regard to the cofactors at the E-site. Although GDP was largely destabilizing in the GDP-Model, GTP becomes relatively largely stabilizing, with an energy change from the GDP-Model of nearly −125±14 kcal/mol. However, this change should not be considered without taking into account the effect of the Mg^2+^ ion that accompanies GTP. This magnesium ion introduces an instability of 95±4 kcal/mol to the GTP-Model. Hence, the overall effect of replacing GDP by GTP and a magnesium ion is a stabilization of 30 kcal/mol on average. Other causes of the lack of stability in the GTP-Model L*β* include the decrease in the contribution of the H11’ helix because GTP offsets interactions by L*β*/His406 (*i* = 1709) by as much as 25 kcal/mol. This is because this histidine is protonated in the GTP-Model and neutral in the GDP-Model and therefore behaves differently in both cases. Being charged in the GTP-Model, it is distracted from the strong attractive vdW interactions it makes with the R*α*/H8-S7 loop by electrostatic and hydrogen bonds with other residues within the L*β* subunit. GTP also causes longitudinal stabilization due to the domains: the H2 helix and the T2 loop to decline while causing stabilization due to the H11-H11’ loop and the T5 loop to rise. As to the R*α*-subunit (Fig 4C), stabilization due to several domains declines in the GTP-Model. These domains include the T7 loop, the S9 strand, the C-terminal loop, the H10 helix, the H12 helix, the H8-S7 loop, and the H10-S9 loop. In short, the GTP-Model is longitudinally less stable than the GDP-Model in most of the domains occurring at the longitudinal inter-dimer interface. An exception to this rule is the increased stabilization due to the C-terminal tail, the N-terminus and the H8 helix, Fig 4C shows the extent of stabilization or destabilization imparted by GTP on each domain. We should also mention that the strong attraction of the R*α*/T7 loop emerging after GTP hydrolysis (Fig 4C) could explain the proposed compaction of the E-site after GTP hydrolysis [15]. In fact, the overall increase in longitudinal dimer-dimer attraction after GTP hydrolysis, as shown by the different values of $E_{tot}^{long}$ in both models, explains the driving force for this E-site compaction.

Among other important residues, R*α*/Lys352 (*i* = 352) of the domain S9 strand has a largely reduced contribution in the GTP-Model, as shown in Fig 5, which is 37 kcal/mol less stabilizing than in the GDP-Model. While having comparable vdW contributions in the two models, this residue suffers strong repulsion probably due to the nearby Mg^2+^ ion in the GTP-Model. Another important residue is R*α*/Val440, located in the C-terminus of the *α*-subunit in our model. GTP enhances the stabilization caused by this residue by nearly 33 kcal/mol over the GDP-Model. Additional important residues and their contributions are shown in the Supporting Information.

### Energy Profile Explains the MT Disassembly Mechanism

**Fig 6:**
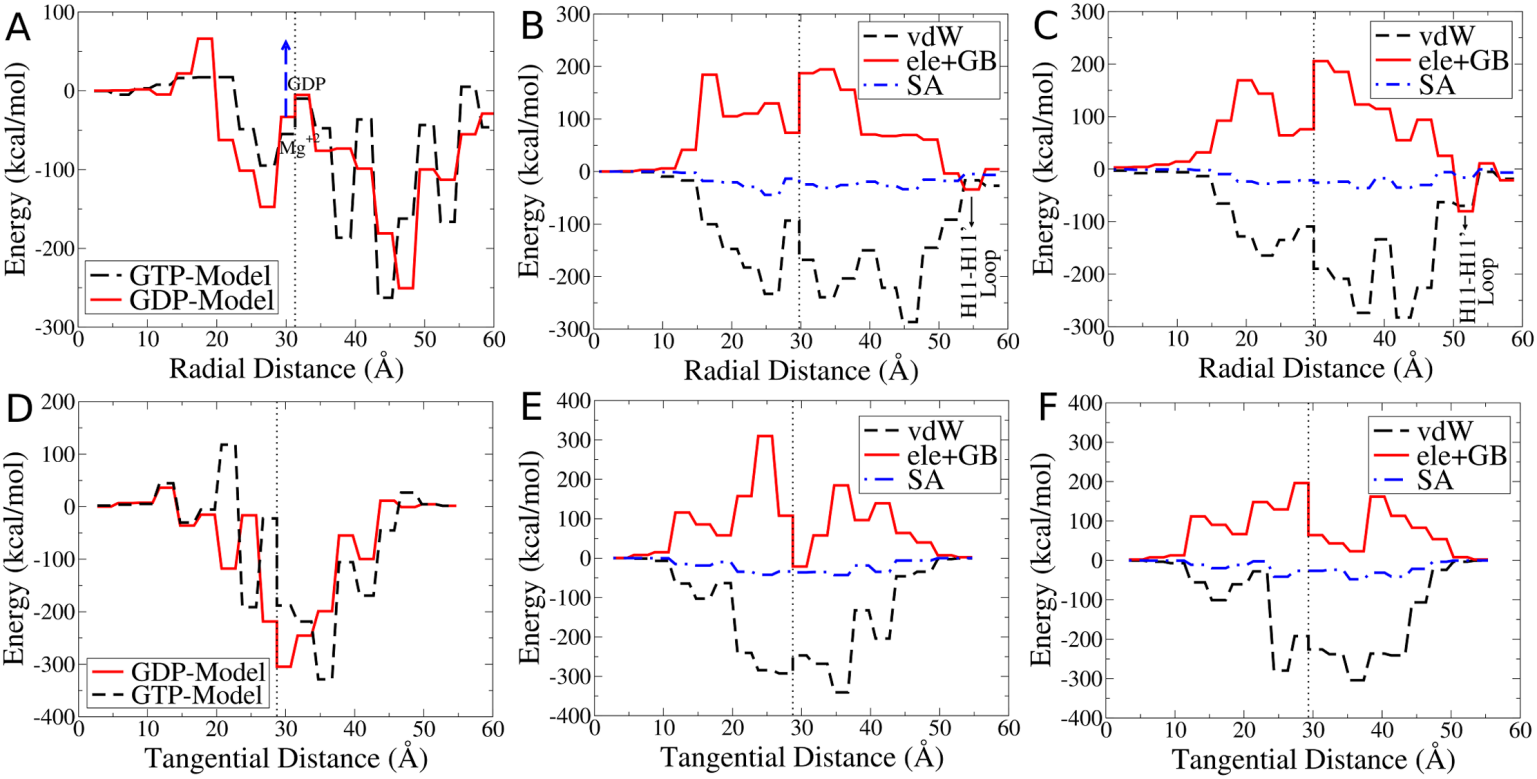
Energy profiles at longitudinal inter-dimer interface. The figures show the sum of energetic contributions of residues located at distance intervals of 3 Å apart, plotted against the radial distance between these residues and the MT lumen (A, B, C) or the tangential distance between the residues and laterally adjacent dimer (D, E, F) in GTP- and GDP-Model. Dotted lines represent the center of mass of tubulin. (A) Radial distribution of total energy in both models. Blue dashed arrow shows how destabilizing the effect of Mg^2+^ is on the GDP-Model if it remains after GTP hydrolysis. (B) Radial distribution of the energy components *E*(vdW), *E*(ele+GB) and *E*(SA) in the GDP-Model and in (C) the GTP-Model, (D) Tangential distribution of total energy in both models. On the *x*-axis, *x* < 30 is the intermediate domain and *x* > 30 is the nucleotide binding domain. (E) Tangential distribution of the energy components *E*(vdW), *E*(ele+GB) and *E*(SA) in the GDP-Model and in (F) the GTP-Model.

Depolymerizing MTs display protofilaments that peel into “ram’s horns” formations under high magnesium buffer conditions. The ends of MTs become frayed, however, under physiological concentrations of magnesium [11]. The energy profile throughout the longitudinal inter-dimer interface provides a clear explanation for the disassembly mechanism, its driving force, and its relation to Mg^2+^ concentration. We characterized each residue in the longitudinal subsystems by its radial distance from the MT lumen in Å, which was plotted on the *x*-axis. The interaction energies of residues, per MT ring, over half-closed intervals of [*x*, *x*+3) were summed up and plotted on the *y*-axis to produce the radial energy profiles in Fig 6A, 6B and 6C.

**Fig 7:**
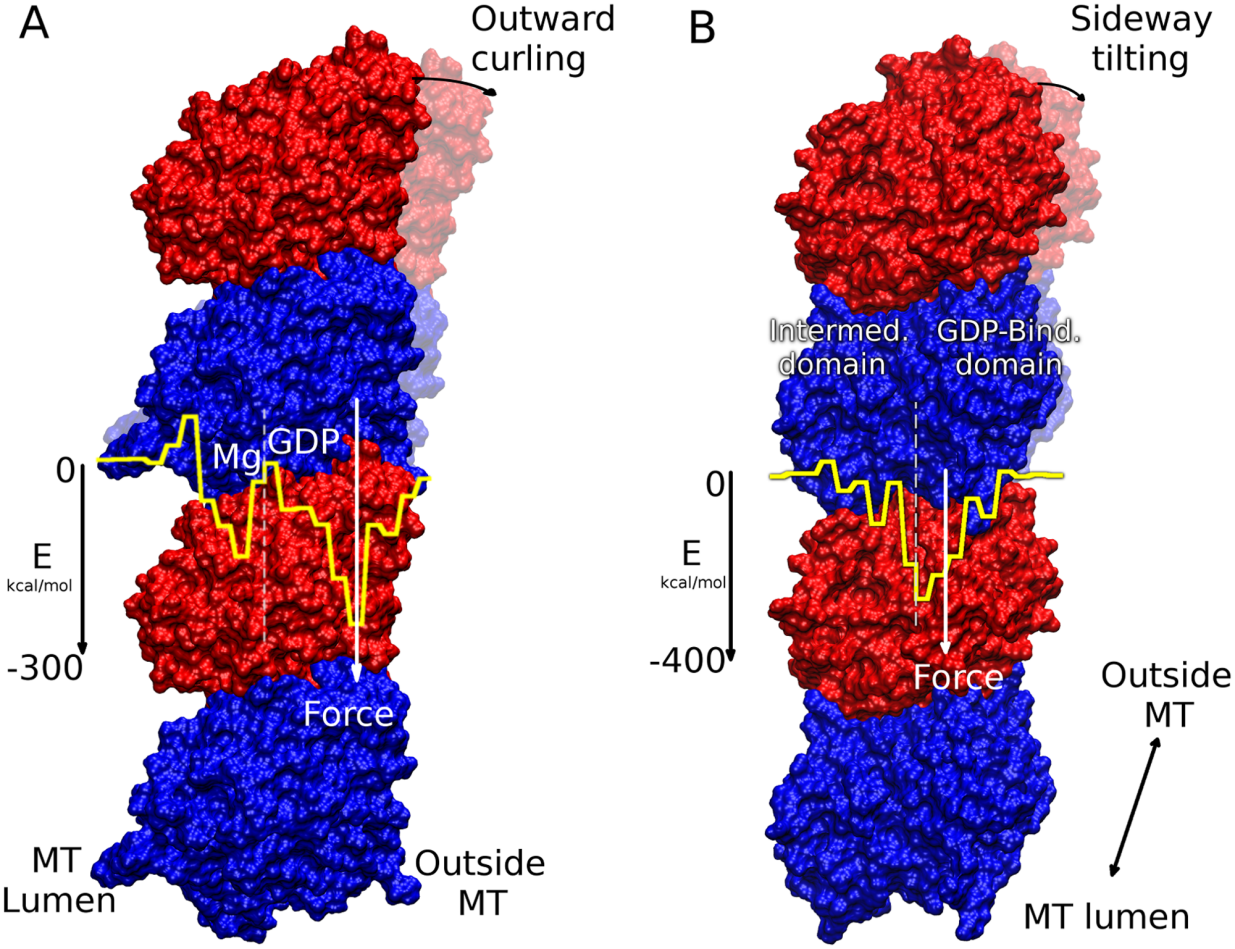
Mechanism of MT disassembly. (A) Radial energy distribution of the GDP-Model at the longitudinal inter-dimer interface is superposed on a protofilament to show how uneven the energy distribution is. This produces a torque that leads to outward curling of the protofilament. (B) Tangential energy distribution of the GDP-Model showing slight sideway tilting due to the slightly uneven distribution of energy. *α*-subunits are colored blue while *β*-subunits are red.

The diagram in Fig 6A leads to a striking observation that the energy distribution throughout the longitudinal inter-dimer interface is not even, with the outward portion (*x* > 30 Å) largely outweighing the inward portion (*x* < 30 Å), with the center of mass of tubulin being at *x* ≈ 30 Å. To mention specific values, in the GTP-Model, the outward portion provides nearly −956 kcal/mol while in the GDP-Model it provides −982 kcal/mol, both values being larger than 80% of the overall longitudinal interaction energy. This uneven distribution of energy, or forces of attraction, is proposed to yield a strong torque that tends to curl MT protofilaments outwardly, breaking lateral bonds and promoting disassembly as illustrated in Fig 7A.

Radial energy profiles of different components of the interaction energy are also shown in Fig 6B and 6C, where electrostatic interactions cause very strong repulsion through the inward portion and attraction only at the periphery where the H11-H11’ loop and particularly residue L*β*/Arg401 are located. We propose a pivotal role for this residue, and for the entire C-terminal domain, in regulating dynamic instability. Electrostatic repulsion by the inner domains and attraction by the outer C-terminal domain is the recipe for outward curling and disassembly in MTs. The vdW distribution will also work, as shown in Fig 6B for the GDP-Model and Fig 6C for the GTP-Model, to curl protofilaments outward until the vdW contacts, and other components, are balanced out.

The largely destabilizing Mg^2+^ ion (see Fig 4D) also plays an important role. Even though GDP at the E-site has low affinity for Mg^2+^ [30], it may still attract Mg^2+^ if it is present in high concentrations or Mg^2+^ may stay in the E-site after GTP hydrolysis. This largely destabilizes the inner portion of the protofilament (blue dashed arrow in Fig 6A), allowing outward forces to pull tubulin out with even less resistance from the other side, thus promoting outward curling and MT disassembly. This explains why large Mg^2+^ concentrations promote ram’s horns formations [31] and increase the rate of disassembly [32, 33], while its low concentrations produce frayed ends and lower rates of disassembly [11].

To explain MT disassembly from a free energy perspective, Fig 7A shows an illustration of the analyzed situation. As already established, uneven distribution of attractive interactions along the longitudinal inter-dimer interface favors outward curling. In the GTP-Model, outward curling is favored by −956 kcal/mol of interaction energy outwardly with respect to the center of mass of tubulin, as compared to −982 kcal/mol in the GDP-Model. These curl-favoring energies/forces are opposed by the lateral interaction energies which tend to pull protofilaments back from both sides, i.e. double the effect. The magnitude of this effect is $2\times E_{tot}^{lat}$, giving −964 kcal/mol in the GTP-Model which is much larger than −822 kcal/mol in the GDP-Model, all energies given per MT ring. We propose that this lateral inward pull balances out the longitudinal outward push in case of the GTP-Model. That being said, the presence of a GTP cap at the tip of the MT would prevent outward curling and thus provide stability for the entire MT structure. After GTP hydrolysis reaches the cap, however, lateral bonds become weaker and longitudinal outward push manages to break the lateral contacts, causing outward curling and MT disassembly. High concentrations of Mg^2+^ may also increase outward curling and the disassembly rate, as explained earlier.

Similar observation could be made about the tangential energy profiles at the longitudinal inter-dimer interface. Fig 6D, 6E and 6F show the tangential energy profiles with the *x*-axis showing the distance from the laterally adjacent protofilament. On the *x*-axis, *x* < 30 is the tubulin intermediate domain while *x* > 30 is the nucleotide binding domain with *x* ≈ 30 being at the center of mass (see Fig 7B). Fig 6D shows that in The GTP-Model, the distribution is also uneven with right-side portion being −1023 kcal/mol (nearly 93% of the total) as compared to −887 kcal/mol (71% of the total) in the GDP-Model. This means that in the GTP-Model, there is a strong force tilting it sideways. However, after GTP hydrolysis and rearrangement of domains at the longitudinal inter-dimer interface, that force largely decreases and the uneven distribution starts to balance out, as shown in Fig 6D, decreasing the strain on lattice integrity. This is in perfect agreement with the recent findings of Alushin et al. [15] They observed that GTP hydrolysis and the release of an inorganic phosphate group leaves a hole within the longitudinal inter-dimer interface between tubulin dimers producing a strain which results in sideway tilting in the same direction [15, 16]. In the present work we show that this tilting is also driven by the uneven energy distribution along the same direction as in the work of Alushin et al. [15] (see Fig 7B). However, this sideway tilting should not be considered as the the driving force for disassembly since it is orthogonal to the outward curling. Combining the two effects together, we conclude that uneven distribution at the longitudinal inter-dimer interface generally leads to a large outward and slight sideway tilting of protofilaments, the former of which is responsible for disassembly of GDP-bound MTs.

### Energy Distribution around the Microtubule Ring

**Fig 8:**
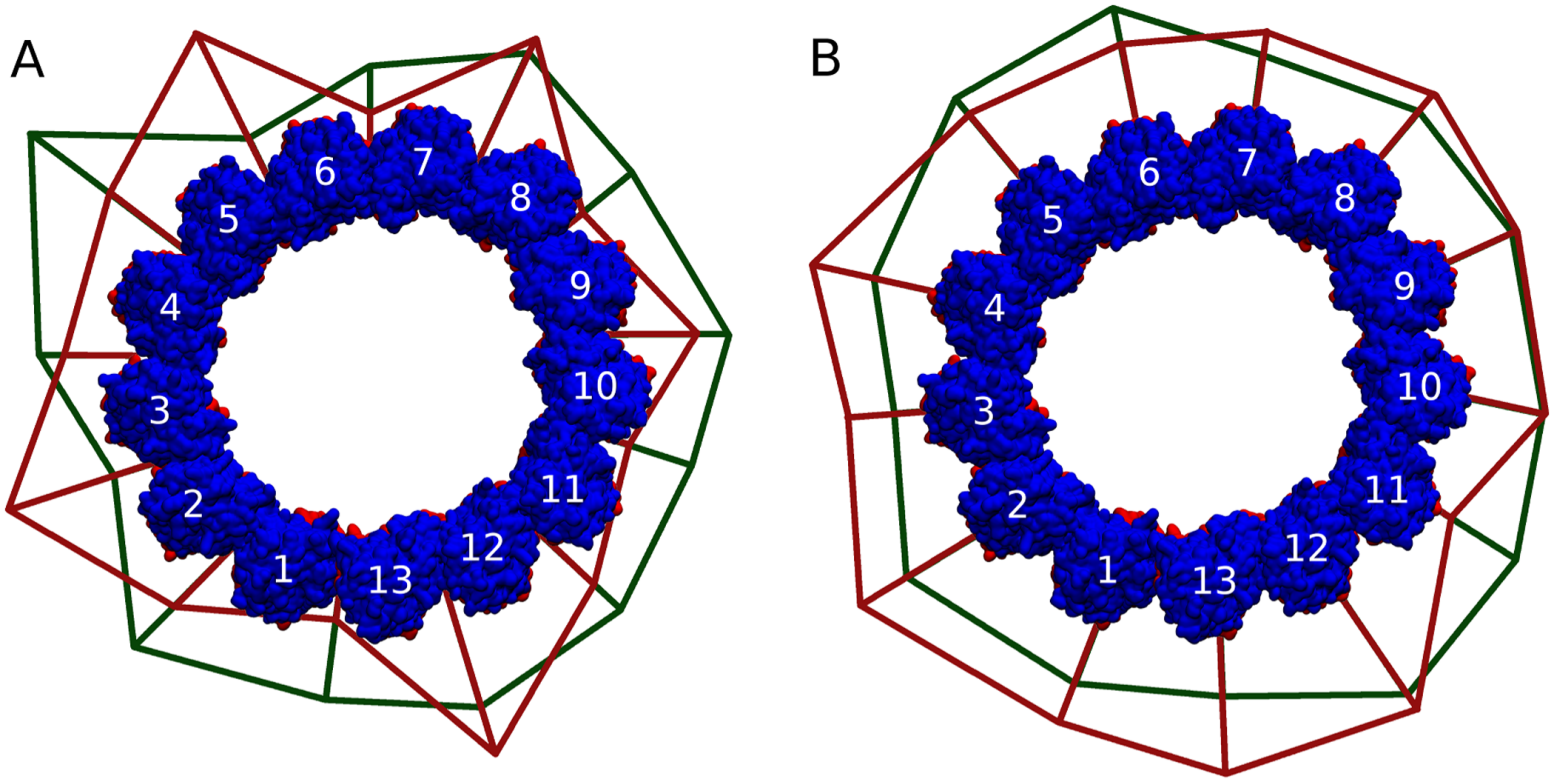
Energy diagrams of the complete MT ring. The diagram shows the magnitude of favorable interaction energies at each interface between two tubulin dimers, whether at (A) the lateral interface, or at (B) the longitudinal inter-dimer interface. The magnitude of the interactions is proportional to the swelling at each interface with swellings in (A) being exaggerated to aid viewing. Green lines represent GTP-Model while red lines represent GDP-Model.

As mentioned in the Methods section, the MT ring was divided into 13 subsystems of laterally adjacent tubulin dimers and another 13 subsystems of longitudinally adjacent tubulin dimers (see Fig 3). All of the energies presented earlier were expressed per MT ring, meaning that they were summed over the 13 subsystems. In this section, however, we focus on the interaction energy in each subsystem. Fig 8A and 8B show energy diagrams for lateral and longitudinal interactions superposed over the MT ring. We first note that the shape of the lateral interactions (Fig 8A) in the GDP-Model is very distorted with several “kinks” of very low energy. When compared to the GTP-Model, its shape is much less distorted. This could come as a straightforward consequence of the fact that GTP-Model is laterally more stable than the GDP-Model and hence suffers less “deformations”.

It is worth mentioning that the deepest of the kinks in the GDP-Model energy diagram, i.e. the interface with the weakest binding energy, is the one occurring at the seam (between dimer 13 and dimer 1), in contrast to its strength in the GTP-Model. It has a binding energy of −9±7 kcal/mol which is very low compared to the one at the interface between dimer 12 and 13, for example, which has an energy of interaction equal to −57±9 kcal/mol. We predict that protofilaments number 1 and 13 having very strong longitudinal contacts antagonized by very weak lateral contacts at the seam, will be the first to dissociate laterally and curve outwards. This should open the MT cylinder which should then trigger disassembly. Therefore, MT energetics suggest that the seam is the most labile inter-dimer interface in the MT structure and could act as a trigger point for disassembly. This is precisely what was reported recently [34].

The energy diagrams at the longitudinal inter-dimer interfaces (Fig 8B) appear to be more even than at the lateral interfaces. However, we see no major difference in the pattern between the GTP-Model and the GDP-Model except that longitudinal interactions in the GDP-Model are stronger, which was established earlier.

## Discussion

We used sophisticated all-atom molecular dynamics simulations to produce accurate MT models, combined with high resolution cryo-electron microscopy maps, to generate an infinite number of infinitely long MT representations. The MM/GBSA energy analysis that followed the simulations enabled an estimate of the contributions of individual residues, domains, subunits and dimers toward the lateral and longitudinal stability of a complete MT ring. We found that longitudinal interactions are about two to three times stronger than lateral interactions explaining the greater stability of the MT structure along its axis than radially. This finding agrees with previous structural observations [28] and computational estimations [18, 22]. We also found that interactions are not evenly distributed radially along the longitudinal inter-dimer interface. That is, attractive interactions are largely concentrated away from the MT lumen, producing a force that curls protofilaments outward and eventually causing MT disassembly. The GTP-Model was laterally more stable than the GDP-Model and the opposite was true for the longitudinal inter-dimer interface. Since lateral forces oppose outward curling while longitudinal forces support it, we expect the GTP-Model to be less prone to disassembly than the GDP-Model. With its lateral forces being strong enough to prevent outward curling caused by longitudinal forces, the GTP-cap at the plus end can stabilize an entire MT cylinder. After GTP hydrolysis reaches the cap, lateral forces are too weak to prevent outward curling, especially at the seam which has the weakest lateral contacts. This results in outward curling and microtubule disassembly.

We also confirmed that the MT seam is most likely to act as a trigger point for MT disassembly by being the most labile interface in the MT cylinder [34]. Magnesium ion was demonstrated to be an influential factor in MT stability. Being present at the inner portion of the longitudinal inter-dimer interface, the largely destabilizing Mg^2+^ ion repels the inward portion and enhances outward curling, the formation of ram’s horns structures and rapid disassembly, which is consistent with key experimental findings [11]. This action of Mg^2+^ at the E-site of tubulin is suppressed by GTP in GTP-capped MTs. As we showed earlier, the ensemble of Mg^2+^ and GTP at the E-site is collectively stabilizing. However, hydrolysis of GTP and release of inorganic phosphate create a gap at the longitudinal inter-dimer interface and leave the largely destabilizing ensemble of GDP and Mg^2+^ which rapidly promotes outward curling to fill this gap. This happens only at large Mg^2+^ concentrations since GDP at the E-site has low affinity for Mg^2+^ [30]. At low Mg^2+^ concentrations, disassembly becomes slower and outward curling becomes less pronounced [11].

Tangential energy profiles at the longitudinal inter-dimer interface were also shown to be uneven and confirmed the hypothesis that GTP hydrolysis produces a strain which promotes sideway titling [15, 16]. However, much of this strain could be tolerated within the lattice constraints and its orthogonality to the direction of outward curling rules out its role in disassembly.

We also identified the most important residues and domains with respect to MT stability at both interfaces and their energetic contributions. At the lateral interface, the *α*/M-loop, *β*/M-loop, *α*/H3 helix, *α*/N-terminal loop and the *α*/H2-S3 loop were shown to be most stabilizing while the *β*/H3 helix was actually destabilizing. This supports predictions based on structural studies [25, 28]. Residue *α*/Tyr283 was shown to form a very strong network of vdW interactions with neighboring residues and to provide the largest amount of stability at the lateral interface. At the longitudinal inter-dimer interface, the *β*/C-terminal domain was found to be of paramount importance not only to stability but also to the mechanism of MT disassembly. In particular, residues *β*/Arg401, *β*/Phe404, and *β*/Trp407 of the C-terminal H11 helix and the H11-H11’ loop were shown to provide more than 20% of longitudinal stability in both the GTP- and GDP-Models. The complete breakdown of MT energetics per every single residue was further analyzed in order to provide crucial insights into many aspects of MT dynamic instability. Of highest importance is the calculation of the amount of force generated through outward curling due to uneven longitudinal interactions. This could help unravel many aspect of the molecular machinery of cell division, in particular the force generation requirement for chromosome segregation.

As a future prospect, simulation of a free protofilament is necessary in order to find out about the effect of uneven longitudinal energy distribution on the extent of outward curling. By comparing the energy of a free protofilament to the energy of a protofilament constrained within our MT model, we can predict the amount of free energy released by outward curling and additional light could be shed on the mechanism and driving forces in MT disassembly. Also, simulating a GDP-Taxol case is necessary to understand the molecular mechanisms by which taxol bound to an MT prevents outward curling and MT disassembly.

## Methods

### Building the Models

The recent structures for GMPCPP and GDP bound MTs at resolutions of 4.7 and 4.9 Å, respectively [15], represented an excellent starting point for building the models presented here. The 3×3 lattice PDB structures of 3J6E (with GMPCPP) and 3J6F (with GDP) were processed using MOE software [35] by the addition of hydrogens and prediction of ionization states. The central tubulin dimer of the 3×3 lattice in each case was separated and was repaired by the addition of missing residues (Residue 1 in *β*-tubulin and residues 1,39 to 48, 440 in *α*-tubulin) from the PDB structure 1TUB [36], using MOE. We modified GMPCPP into GTP since in our simulations there is no need to use the nonhydrolyzable GTP analogue as hydrolysis is not expected in MD simulations. Next, for both GTP and GDP systems, the repaired tubulin was superimposed over the 13 tubulin dimers in the complete MT model built by Wells and Aksimentiev [24], producing a hybrid complete MT model for both systems. Thus, we produced two models, the GTP-Model and the GDP-Model, by combining the helical structural configuration developed by Wells and Aksimentiev with the lattice tubulin coordinates obtained from Alushin’s model. Several clashes existed at lateral interfaces between tubulin dimers and were resolved through a short minimization using the Generalized Born (GB) continuum model in Amber [37].

Each model, as shown in Fig 1B, has 13 tubulin dimers in an MT orientation. For the GDP-Model, each tubulin has GTP, Mg^2+^ and four coordinating water molecules at the *α*-tubulin N-site, and GDP at the *β*-tubulin E-site. For the GTP model, there was GTP, Mg^2+^ and four coordinating water molecules at both the N-site and the E-site. Solvation was carried out using box of dimensions 293.85 × 293.85 × 83.38 (or 81.20) Å^3^ for the GTP- and GDP-Models, respectively. The *z*-component was obtained from Alushin’s lattice structure [15] and ensures perfect longitudinal alignment of tubulin dimers in both systems (see Fig 1B). Both *x* and *y* components were obtained from Wells’ structure [24]. A total of 181,000 TIP3P water molecules were added in the solvation box. This number was obtained based on several optimization trials which guaranteed consistency in box dimensions and density throughout the simulations. A total number of 442 Na^+^ ions was needed for neutralizing the GTP-Model, versus 455 for the GDP-Model. An extra 327 Na^+^ and Cl^−^ ions were added to bring the salt concentration to 0.1 M.

During the addition of water and ions, we made sure that no atoms were placed in positions which will be occupied by the periodic images of our system in both the positive and negative *z* direction (see the gaps in the water box of Fig 1B). Thus, exploiting the periodic boundary conditions, the mirroring of our nearly 720,000-atom system in all directions should effectively result in an infinite number of infinitely long MTs, (see S1 Movie). The AMBER Molecular Dynamics package was used for solvation, ionization, and dynamics [37].

### Parameterization and Dynamics

The all-atom forcefield AMBERff12SB was used to parameterize the protein [38, 39]. Cofactors were parameterized utilizing the parameter set developed by Meagher et al. [40]. Each of the two systems was then minimized through nearly 1000 steps of the steepest descent algorithm followed by about 6000 steps of the conjugate gradient algorithm. Then, the systems were heated, with restraints of 10 kcal mol^−1^ Å^−2^ on the protein, to a temperature of 310 K using the Langevin thermostat over 20 ps under constant volume. This was followed by 200 ps of density equilibration under constant temperature and pressure, in which the restraints were eliminated gradually, followed by a production phase of 50 ns for each system. Simulations were performed using NVIDIA Tesla K20X GPU cards on the PharmaMatrix Cluster (University of Alberta) through AMBER GPU-accelerated code [41–43]. All simulations were performed using periodic boundary conditions employing the particle-mesh Ewald method for treating long-range electrostatics and a non-bonded cut off of 10.0 Å under constant pressure with anisotropic pressure scaling.

### Trajectory Analysis

The 50-ns trajectory of each system was analyzed for several structural and conformational aspects. Most of the analysis was done utilizing the CPPTRAJ module in AMBER [44], MM/GBSA implementation in AMBER [45] plus several scripts that we designed to facilitate data analysis. The software VMD 1.9.1 was also used for viewing trajectories and image rendering [46].

Data analysis included calculating the total as well as the per-residue MM/GBSA binding energies [47] between pairs of tubulin dimers in lateral and longitudinal orientations. These calculations involved all the 13 heterodimers included in the simulations and would always give the energy per MT ring (Fig 1B). Hence, energetic contributions were assessed via the equation:

$$\begin{array}{c} E_{x}=\epsilon_{x}\left(R_{13}L_{1}^{\prime }\right)+\sum_{k=1}^{12}\epsilon_{x}\left(R_{k}L_{k+1}\right) \end{array}$$ (1)

for lateral systems, and the equation:

$$\begin{array}{c} E_{x}=\sum_{k=1}^{13}\epsilon_{x}\left(R_{k}L_{k}^{\prime }\right) \end{array}$$ (2)

for longitudinal systems. In both equations, *E*_*x*_ represents an energetic contribution of a given residue, domain or subunit *x* per MT ring of 13 tubulin dimers shown in Fig 1B. In Eq 1, *ϵ*_*x*_(*R*_*k*_ *L*_*k*+1_) is the energetic contribution of the same entity *x* in a subsystem composed of only tubulin *k*, treated as a “receptor”, and tubulin *k*+1, treated as a “ligand”. $\epsilon_{x}(R_{13}L_{1}^{\prime })$ does the same but at the lateral seam, taking into account the flip between *α*- and *β*-subunits. In Eq 2, $\epsilon_{x}(R_{k}L_{k}^{\prime })$ carries the same concept except that the ligand in a longitudinal subsystem is simply the periodic image of the receptor, hence the prime. Therefore, we ended up investigating 12 lateral subsystems plus 1 lateral subsystem at the seam and 13 longitudinal subsystems, for each model. An illustration of each subsystem is shown in Fig 3. Hence, our convention in this work is that the dimer whose M-loop is involved in lateral interactions is always termed “receptor” in lateral subsystems, and the dimer whose *α*-tubulin is involved in longitudinal interactions is always termed “receptor” in longitudinal subsystems. This distinction was necessary since we noticed that energetic contributions can vary between tubulin dimers acting as receptors and those acting as ligands.

All the energy calculations were performed on 200 evenly-spaced snapshots from the last 10 ns of the molecular dynamics trajectory where equilibration was confirmed. A solvent and solute dielectric constant of 80 and 1, respectively, were used for electrostatics in the Amber MM/GBSA implementation.

## Supporting Information

S1 Dataset Lateral GDP Energetics. 13 text files with energy contribution per residue in the 13 lateral GDP subsystems, plus one text file with the overall residual contributions, per MT ring.
(ZIP)

S2 Dataset Lateral GTP Energetics. 13 text files with energy contribution per residue in the 13 lateral GTP subsystems, plus one text file with the overall residual contributions, per MT ring.
(ZIP)

S3 Dataset Longitudinal GDP Energetics. 13 text files with energy contribution per residue in the 13 longitudinal GDP subsystems, plus one text file with the overall residual contributions, per MT ring.
(ZIP)

S4 Dataset Longitudinal GTP Energetics. 13 text files with energy contribution per residue in the 13 longitudinal GTP subsystems, plus one text file with the overall residual contributions, per MT ring.
(ZIP)

S1 Movie Model Construction. A movie showing the model construction and the effect of periodic boundary conditions.
(MP4)

S2 Movie GDP-Model Diameter Change. A movie showing the change of the two perpendicular diameters (in Å) in the GDP-Model over simulation time.
(MP4)

S3 Movie GTP-Model Diameter Change. A movie showing the change of the two perpendicular diameters (in Å) in the GTP-Model over simulation time.
(MP4)
